# Telomerase regulation by the Pif1 helicase: a length-dependent effect?

**DOI:** 10.1007/s00294-017-0768-6

**Published:** 2017-10-20

**Authors:** Sonia Stinus, Katrin Paeschke, Michael Chang

**Affiliations:** European Research Institute for the Biology of Ageing, University Medical Center Groningen, University of Groningen, A. Deusinglaan 1, 9713 AV Groningen, The Netherlands

**Keywords:** Pif1, Telomeres, Telomerase, Telomere length homeostasis, Double-strand breaks

## Abstract

Dysfunctional telomere length regulation is detrimental to human health, and both activation and inhibition of telomerase have been proposed in potential therapies to treat human diseases. The *Saccharomyces cerevisiae* Pif1 protein is an evolutionarily conserved helicase that inhibits telomerase activity at DNA ends. Recent studies have indicated that Pif1 is specifically important for inhibiting telomerase at DNA ends with very little or no telomeric sequence and at long telomeres. At the former, Pif1 prevents the inappropriate addition of a telomere at DNA double-strand breaks. For the latter, Pif1 has been shown to bind long telomeres to presumably promote the extension of the short ones. These observations leave the impression that Pif1 does not act at DNA ends with telomeric sequence of intermediate length. Here, we provide in vivo evidence that Pif1 inhibits telomerase activity at DNA ends regardless of telomere sequence length.

The ends of eukaryotic chromosomes are capped by telomeres, nucleoprotein complexes that protect chromosome ends from degradation, and telomere–telomere fusion (Ferreira et al. 2004). Telomeres can shorten due to incomplete replication or nuclease-dependent degradation. This shortening is counteracted by a specialized reverse transcriptase called telomerase (Greider and Blackburn 1985). The consequences of improper telomere length regulation are severe: several human diseases are linked to telomere shortening (reviewed in Blasco 2005; Armanios and Blackburn 2012), and upregulation of telomerase is a common feature in cancer cells (Kim et al. 1994).

Telomere length homeostasis maintenance is a tightly regulated process and has been best studied in the budding yeast *Saccharomyces cerevisiae*, whose telomeres consist of 300 ± 75 bp of C_1–3_A/TG_1–3_ repeats (Wellinger and Zakian 2012). Not all telomeres are elongated during each cell cycle and the probability of extension of a given telomere is inversely correlated to its length, with shorter telomeres preferentially elongated (Teixeira et al. 2004). In budding yeast, the double-stranded telomeric DNA-binding protein Rap1 forms a complex together with Rif1 and Rif2 to negatively regulate telomerase (Wotton and Shore 1997). According to the protein-counting model (Marcand et al. 1997), telomere-bound Rap1 increases proportionally with telomere length, preventing telomerase from extending the longer telomeres, thereby providing a mechanism to maintain telomere length homeostasis.

The evolutionarily conserved Pif1 helicase is also an important player in telomere length regulation. In vitro, Pif1 reduces the nucleotide addition processivity of telomerase, removes telomerase from telomere-like oligonucleotides (Boulé et al. 2005), and unwinds the telomere–telomerase complex (Li et al. 2014). In vivo, mutation of *PIF1* causes telomere lengthening (Schulz and Zakian 1994) and increases telomere association of the telomerase subunits, Est1 and Est2 (Boulé et al. 2005; Phillips et al. 2015). On the other hand, Pif1 overexpression has been shown to cause telomere shortening (Zhou et al. 2000) and to reduce Est1 and Est2 binding to chromosome ends (Boulé et al. 2005). Taken together, the evidence suggests that Pif1 regulates telomere length by unwinding the DNA–RNA hybrid formed by the telomeric DNA and telomerase RNA subunit.

Besides telomere length regulation, Pif1 function is also important at double-strand DNA breaks (DSBs). Gross chromosomal rearrangements (GCR) are known to be increased 230-fold in *pif1-m2* cells, where only the nuclear function and not the mitochondrial function of Pif1 is affected (Schulz and Zakian 1994), and 1000-fold in *pif1∆* cells (Myung et al. 2001). The increase of GCR events in *pif1-m2* cells is due to improper and deleterious addition of telomere sequence, and deletion of telomerase subunits rescues the GCR rate to wild-type levels (Myung et al. 2001). Pif1 is thought to prevent telomere sequence addition at DSBs in cooperation with Mec1-dependent inhibition of the single-stranded telomeric DNA-binding protein Cdc13, which is needed for the recruitment of telomerase (Nugent et al. 1996). In response to DNA damage, Cdc13 and Pif1 are phosphorylated in a Mec1-dependent manner to preserve genome stability. Phosphorylation of Cdc13 inhibits its accumulation at DSBs, thus preventing telomerase recruitment to these sites, and phosphorylated Pif1 removes telomerase from DSBs (Makovets and Blackburn 2009; Zhang and Durocher 2010).

Remarkably, although Pif1 functions both at DSBs and at telomeres, it is differently regulated at these two DNA ends. Two phosphorylation mutants of Pif1 support this idea: the unphosphorylatable *pif1-4A* mutant (T763A/S765A/S766A/S769A) is unable to inhibit telomere addition at DSBs, but it does not result in bulk telomere lengthening at chromosome ends, whereas the *pif1-4D* mutant, which mimics constitutive phosphorylation, restores Pif1 activity at DSBs but does not give rise to shorter telomeres. This observation implies that phosphorylation of Pif1 is important for inhibiting telomerase at DSBs and not at telomeres (Makovets and Blackburn 2009).

In a recent study, Pif1 was reported to be found preferentially at long telomeres, and it was suggested that this serves to promote telomerase-dependent extension of short telomeres (Phillips et al. 2015). We have also recently published a study where the inducible HO cut system was used to create DNA ends adjacent to telomeric sequence of different length to monitor the ability of Pif1 to inhibit telomerase. We found that a 34-bp telomere tract is enough to render such an end insensitive to Pif1, resulting in a very high frequency of telomere addition. We defined this tract length as the DSB-telomere threshold, below which Pif1 actively supresses telomere addition (Strecker et al. 2017). This result suggests that Pif1 activity becomes irrelevant at telomeres longer than 34 bp.

Together, the two above-mentioned studies give the impression that Pif1 activity is only relevant in two scenarios: (1) at DNA ends with no telomeric sequence or with telomeric sequence shorter than the DSB-telomere threshold length, where its telomerase inhibitory activity protects genome stability by preventing inappropriate addition of telomeric sequences at DSBs, and (2) at long telomeres, where Pif1 inhibits telomerase to facilitate the extension of short telomeres. However, our data show that the telomerase inhibitory activity of Pif1 takes place at all DNA ends, regardless of telomere length, and it is, therefore, telomere length independent.

We monitored telomerase-dependent telomere extension events in wild-type and *pif1-m2* cells using the iSTEX assay, which allows us to detect telomere extension events at individual telomeres at nucleotide resolution during a single cell cycle (Strecker et al. 2017). In wild-type cells, as previously reported, the frequency of telomere extension increases as telomere length decreases, and there is an increase in the extent of elongation at telomeres below 125 nt (Teixeira et al. 2004). In *pif1-m2* cells, the extent of elongation at telomeres below 125 nt is even greater (Fig. 1a, b). Yeast telomerase is generally non-processive in terms of repeat addition, except at telomeres less than 125 nt in length, where an increase in repeat addition processivity leads to an increase in extension length (Chang et al. 2007). It is not known how this increased processivity is achieved, although it is known to be dependent on Tel1 (Chang et al. 2007), and we now propose that it is also inhibited by Pif1. The extent of elongation is not significantly different at telomeres greater than 125 nt in *pif1-m2* cells, indicating that the lack of repeat addition processivity at such telomeres is not due to the presence of Pif1. Furthermore, we found an increase in the frequency of extension at telomeres of all length, but short telomeres were still preferentially extended (Fig. 2a, b). This implies that telomerase activity is inhibited by Pif1 in a telomere length-independent fashion.

**Fig. 1:**
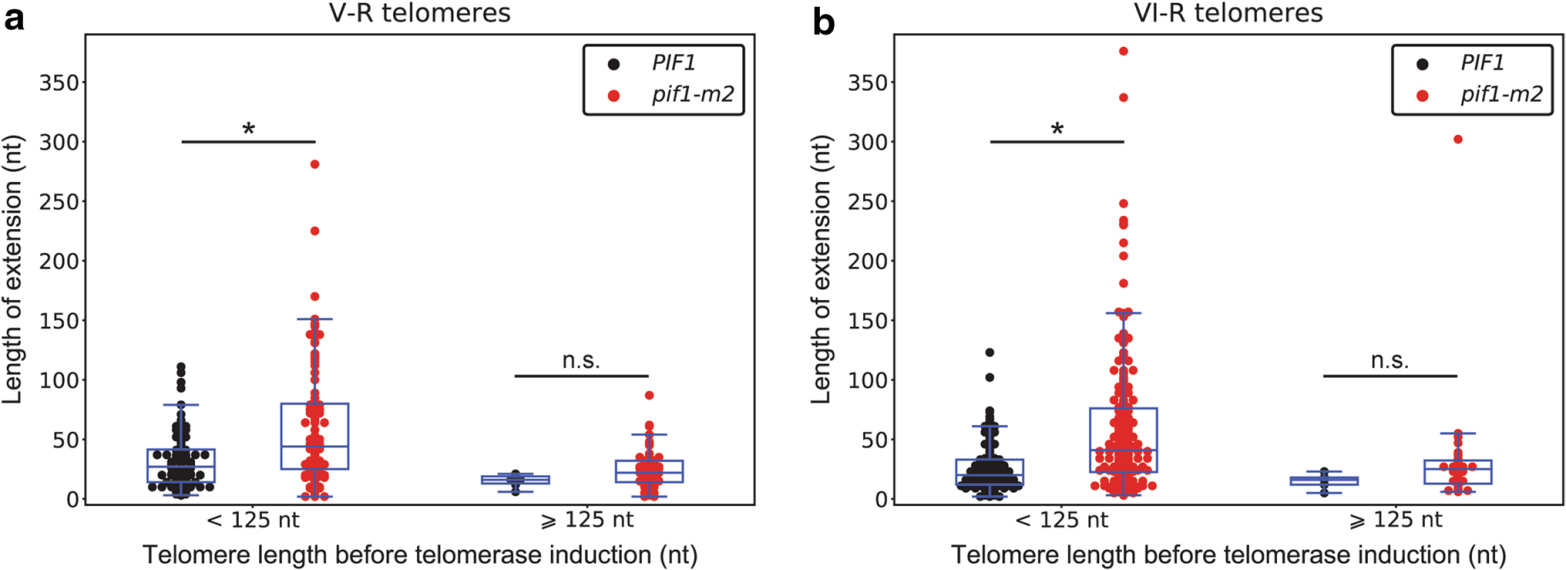
Mutation of *PIF1* increases telomere extension length at telomeres less than 125 nt in length. Length of telomere extension for telomeres V-R (**a**) and VI-R (**b**), obtained from iSTEX analysis of wild-type and *pif1-m2* cells (Strecker et al. 2017), is plotted according to telomere length prior to telomerase induction (< 125 and ≥ 125 nt). Statistical significance was determined using a two-tailed Mann–Whitney *U* test. *p value < 0.00001 and *ns* not significant

**Fig. 2:**
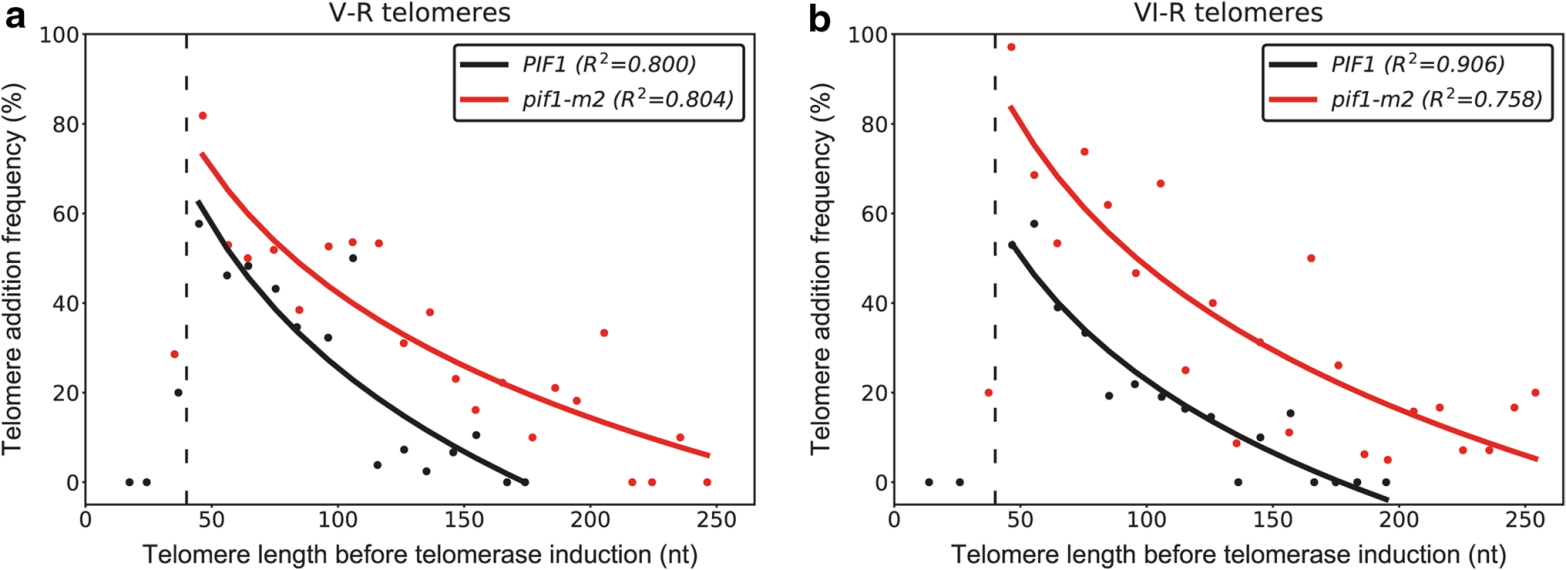
Mutation of *PIF1* increases the frequency of telomere extension. Telomere V-R (**a**) and telomere VI-R (**b**) sequences obtained from the iSTEX analysis of wild-type and *pif1-m2* cells (Strecker et al. 2017) were binned into groups of 10 nt in size according to telomere length before telomerase induction. Groups containing less than five telomeres were excluded from this analysis. Frequency of extension and average telomere length before telomerase induction were calculated and plotted for each group. Logarithmic regression curves for each data set were determined using Microsoft Excel. The equations of the curves for telomere V-R are *y* = − 46.06ln(*x*) + 237.61 (wild-type) and *y* = − 40.13ln(*x*) + 227.05 (*pif1-m2*). The equations of the curves for telomere VI-R are *y* = − 39.89ln(*x*) + 206.47 (wild-type) and *y* = − 46ln(*x*) + 259.99 (*pif1-m2*). *R* ^2^ indicates coefficient of determination. Telomeres shorter than 40 nt before telomerase induction, below the DSB-telomere threshold (dashed line), were excluded from the regression analysis

This finding is in agreement with a previous study showing that the frequency and extent of elongation of telomeres between 50 and 200 nt in length was increased in *pif1∆* cells (Phillips et al. 2015). The same study also reported increased Est1 and Est2 binding to an HO-induced TG_80_ end, which mimics a short telomere, in *pif1-m2* cells compared to the wild-type control. Intriguingly, Pif1 was also found to preferentially bind long telomeres (Phillips et al. 2015). If Pif1-mediated inhibition of telomerase is independent of telomere length, then why is Pif1 preferentially bound to long telomeres? Pif1 has several other functions in addition to telomerase inhibition. Pif1 is also known to function in Okazaki fragment processing (Budd et al. 2006) and to promote replication of hard-to-replicate sites, such as telomeres (Paeschke et al. 2011). Thus, the interpretation of Pif1 binding to telomeres is complicated by the telomerase-independent functions of Pif1 at telomeres. Longer telomeres would mean more DNA to be replicated, and may, therefore, require more Pif1.

A telomere length-independent regulation of telomerase activity by Pif1 is also in conflict with the idea that Pif1 is active only below the DSB-telomere threshold to avoid telomere sequence addition at DSBs (Strecker et al. 2017). However, the genetic assay to monitor telomere addition (which measures the ability of telomerase to add a telomere to a DNA end generated by the HO endonuclease, thereby allowing the cell to grow) might be less sensitive to detect Pif1 activity as compared to the iSTEX assay. While the iSTEX assay detects telomerase-mediated extension events during a 2-h window, the inducible HO method allows a much longer time for a telomere addition event to occur: telomere addition can take place at any time during the 4-h growth period in galactose-containing medium to induce the HO cut, and within a few hours after plating, which would still give enough time for a cell to add a telomere to the DSB and form a colony at the end of the 3-day experiment. Therefore, telomere addition frequency in the inducible HO cut method is already very high at a DNA end adjacent to telomeric sequence above the DSB-telomere threshold (~ 90% at a DNA end with 34 bp of telomeric sequence; Strecker et al. 2017), making a further increase in frequency undetectable upon mutation of *PIF1*.

In summary, we have found that Pif1 inhibits telomerase in a similar way across all telomere lengths, indicating that its function on telomerase inhibition does not depend on telomere length. However, several questions remain unanswered. How does phosphorylation of Pif1 only affect its activity at DNA ends below the DSB-telomere threshold? If Pif1 is acting on both sides of this threshold, how is the threshold set? As we previously proposed (Strecker et al. 2017), a DNA end below the DSB-telomere threshold is unable to efficiently recruit or activate telomerase for a still unclear reason, and Pif1 activity ensures that telomerase is tightly inhibited. Above the threshold, telomerase recruitment/activation is strong, making Pif1 largely irrelevant. The difference above and below the threshold is independent of the interaction between Cdc13 and telomerase (Strecker et al. 2017), so, perhaps, it is recruitment of Cdc13 itself that is important. Furthermore, characterizing the role of Pif1 in telomerase inhibition is complicated by the telomerase-independent functions of Pif1, which could indirectly affect telomerase activity. Most of its telomerase-independent functions—Okazaki fragment processing, DNA end resection, break-induced replication, G-quadruplex unwinding, and destabilization of R-loops—are either known to or have been proposed to occur at telomeres as well (Dewar and Lydall 2010; Geronimo and Zakian 2016). Further work will be required to characterize what role Pif1 plays in each of these processes at telomeres.

Pif1 helicases are highly conserved and its family members are identified from bacteria to humans (Bochman et al. 2010). Human Pif1, which has 24% sequence identify to Pif1 in *S. cerevisiae* (Bochman et al. 2010), was shown to bind to telomeric sequences in vitro (Zhang et al. 2006) and to interact with the catalytic subunit of telomerase (Mateyak and Zakian 2006). These observations, together with the fact that telomerase is upregulated in ~ 90% of human cancers (Hanahan and Weinberg 2011), make Pif1 and its associated proteins possible targets for future therapies involving the alteration of telomerase activity.
